# The role of vasopressin in trauma resuscitation: a protocol of a systematic review and meta-analysis of randomized and observational studies

**DOI:** 10.1186/s13049-026-01581-w

**Published:** 2026-02-11

**Authors:** Mohammad Reza Yousefi, Mehdi Ghasemian, Shahram Paydar

**Affiliations:** Trauma Research Center, Shiraz University of Medical Sciences, Shiraz, Iran

**Keywords:** Injury, Trauma, Hemorrhagic shock, Resuscitation, Vasopressin, Anti diuretic hormone

## Abstract

**Introduction:**

Hemorrhagic shock caused by trauma is one of the most important contributors to mortality in severely injured patients. Management traditionally includes hemorrhage control and aggressive fluid resuscitation. Severe hemorrhagic shock induces a state of antidiuretic hormone (vasopressin) deficiency, which exacerbates vasodilatory shock and refractory hypotension. Recent clinical trials and reviews emphasize the potential benefits of low-dose vasopressin supplementation during trauma resuscitation. Vasopressin can restore vascular tone by acting independently of the adrenergic system, improve renal perfusion, promote hemostasis, and reduce the volume of required blood product transfusions without increasing complications. In this study, we aim to review the effect of AVP in traumatic patients presenting with hemorrhagic shock.

**Methods:**

This review is formatted according to the Preferred Reporting Items for Systematic Review and Meta-Analysis Protocols (PRISMA-P). Quantitative randomized and observational study designs that evaluated the administration of vasopressin in trauma and hemorrhagic shock will be considered for systematic review. Databases PubMed (NCBI), PubMed Central (NCBI), Embase (Ovid), Scopus (Elsevier), and ClinicalTrials.gov (NLM) were searched for articles from 2000 to September 2025. Cochrane Library (Wiley) is also reviewed for possible systematic reviews on the subject. Further articles are sought through forward and backward citation tracking of the articles with the highest impact, especially randomized trials. Two independent blinded reviewers will screen the remaining articles for eligibility and methodological quality. Two independent authors will extract the data to decrease errors and bias. For statistical analysis, effect sizes will be expressed as risk ratios or ORs for dichotomous data or weighted (or standardized) mean differences and 95% CIs for continuous data.

**Trial registration:**

CRD420251154486. https://www.crd.york.ac.uk/PROSPERO/view/CRD420251154486

**Supplementary Information:**

The online version contains supplementary material available at 10.1186/s13049-026-01581-w.

## Introduction

Trauma remains a leading cause of death worldwide, with hemorrhagic shock being a primary contributor to early mortality in severely injured patients [1–4]. Management traditionally centers around hemorrhage control and aggressive fluid resuscitation, yet this approach carries risks such as coagulopathy, tissue edema, and organ dysfunction. Notably, severe hemorrhagic shock induces a state of arginine vasopressin (AVP) deficiency, which exacerbates vasodilatory shock and refractory hypotension [5]. It is notable, however, that recent studies show a clear move from crystalloid‑heavy resuscitation to early, balanced blood product or whole blood strategies in hemorrhagic trauma [6, 7].

AVP is a peptide hormone produced by the hypothalamus and released by the posterior pituitary gland. It plays a significant role in regulating water balance, blood pressure, and, in certain clinical contexts, bleeding [8]. Vasopressin exerts its effects primarily through V1 and V2 receptors. V1 receptors mediate vasoconstriction, which can elevate blood pressure, while V2 receptors promote water reabsorption in the kidneys, concentrating urine and reducing blood volume [9].

Recent clinical trials and reviews emphasize the potential benefits of low-dose AVP supplementation during trauma resuscitation. Studies indicate that exogenous AVP can effectively restore vascular tone by acting independently of the adrenergic system, improve renal perfusion, promote hemostasis, and reduce the volume of required blood product transfusions without increasing complications.

Based on recent evidence, arginine vasopressin (AVP) enhances platelet aggregation and hemostasis in hemorrhagic shock, primarily by stimulating V1a receptors on platelets to increase their procoagulant activity, promote the exocytosis of von Willebrand factor from endothelial cells, and augment platelet-dependent thrombin generation—mechanisms that improve clot formation and hemorrhage control, supporting a potential role as an adjunctive agent in trauma resuscitation [10]. While AVP's pro-hemostatic mechanisms raise theoretical concerns about tipping the coagulation balance toward pathologic thrombosis, clinical trial data do not demonstrate increased DVT, pulmonary embolism, myocardial infarction, or ischemic stroke with physiologic dosing (≤ 0.04 U/min) [11].

While animal and clinical studies support AVP as a promising adjunct therapy, its use in traumatic hemorrhagic shock is somewhat surrounded by controversies [12]. A previous retrospective study on a cohort of trauma patients who required vasopressors within 72 h of their admission demonstrated a higher mortality rate among the patients receiving AVP, despite their higher probability of survival based on injury severity scores [13]. On the other hand, some studies have indicated lower fluid and blood product requirements in trauma patients [10, 14]. Therefore, further investigation to define optimal dosing, timing, and overall impact on morbidity and mortality appears to be necessary.

In conclusion, the potential role of AVP in decompensated hemorrhagic shock [15] can position AVP as a potentially valuable tool in trauma care to mitigate resuscitation-associated risks and improve patient outcomes, most notably, as a therapy of last resort [16, 17]. In light of these findings, we aimed to review the effect of AVP on the mortality and fluid requirements of traumatic patients presenting with hemorrhagic shock.

## Methods

### Protocol and registration

This review is formatted according to the Preferred Reporting Items for Systematic Review and Meta-Analysis Protocols (PRISMA-P) 2015 statement [18]. It is also registered in the International Prospective Register of Systematic Reviews (PROSPERO) with registration number: CRD420251154486. The proposed study will be reported consistently with the Preferred Reporting Items for Systematic Reviews and Meta-Analyses (PRISMA) statement [19] (Supplement Appendix 3).

### Eligibility criteria

This study will cover original studies on adult trauma patients with hemorrhagic shock and possible need for resuscitation, regardless of the cause of injury. Studies that incorporated any form of trauma that required resuscitation and possible blood product transfusion, due to severe blood loss or hemorrhagic shock, are considered for review. The administration of anti-diuretic hormone (ADH), also known as vasopressin, is the key variable of our review. Quantitative randomized and observational study designs that evaluated the administration of vasopressin in comparison with the use of routine resuscitation drugs or no drugs at all will be considered for systematic review. Only studies with adequate quantitative data reporting that can be used in calculating ORs and effect sizes will be enrolled in the review. All the different dosage ranges and administration methods are evaluated. This review will include all studies that evaluated mortality, hemodynamic stability, transfusion needs, and adverse events in the study population. Studies with inadequate data reporting and insufficient details regarding study design, as well as narrative reviews, letters, case reports, and articles without available full text are excluded. Furthermore, incomplete trials, retracted articles, and articles not in the English language will not be considered for this review.

### Information sources and search strategy

Databases PubMed (NCBI), PubMed Central (NCBI), Embase (Ovid), Scopus (Elsevier), and ClinicalTrials.gov (NLM) are searched for articles from 2000 to September 2025. Cochrane Library (Wiley) is also reviewed for possible systematic reviews on the subject. Further articles are sought through forward and backward citation tracking of the articles with the highest impact, especially randomized trials [10, 14, 16, 20], using Paperfetcher online services [21] and citation lists. The details of search strategies and yielded articles from each source are shown in the supplement appendix 1. Notably, grey literature will not be included in the proposed review.

### Study selection

All the acquired articles will be uploaded to the Rayyan webapp for systematic review [22], and duplicated articles will be deleted under the supervision of an author (MRY). Thereafter, two independent blinded reviewers (MRY and MG) will screen the remaining articles for eligibility in three steps: 1) screening the article titles and excluding inappropriate ones; 2) the abstracts of the remaining articles will be reviewed for eligibility of the reporting data; 3) the full text of the included articles will be thoroughly examined. If any disagreement arises, a third reviewer (SP) will settle the argument. Finally, a PRISMA flow-diagram will be designed to summarize the process and specify the reason for any full-text exclusion.

### Data collection and outcomes

The PRISMA guide for data extraction will be used by two authors (MRY and MG) to gather data from the selected articles. Those authors will review the articles independently to minimize the chance of bias and improve the accuracy of the analysis; if any disagreement arises, the authors will sort it out with discussion among themselves or the with third author (SP). The extracted data will be established upon the primary outcome (mortality rate) and secondary outcomes (fluid and blood products requirement, as well as adverse effects) and include first author’s name, year of study, type of study, sample size, country of data origin, demographic characteristics of the study population, severity of injury, in-hospital mortality rate, 30-days mortality rate, type of resuscitation drugs, dosage of administered vasopressin, Co-interventions that could affect mortality (e.g., need for blood products transfusion, tranexamic acid administration, REBOA, etc.), effect measures reported (RR, OR, HR), and adverse drug reactions (see supplement appendix 2 for more details). If sufficient data are available, predefined subgroup analyses may be conducted based on clinically relevant variables, including timing of vasopressin administration (early vs late), study design (randomized vs observational), and type of trauma context. Where data are insufficient, these factors will be explored descriptively.

### Assessment of risk of bias in individual studies

Two independent authors will strictly review the methodology and protocols of the studies and assess the quality with RoB 2 and ROBINS-I version II tools for randomized and non-randomized studies, respectively [23, 24]. Any study with low methodological quality that does not meet the required quality level will be excluded from the review, and a reason will be disclosed. Any disagreement between the two authors will be resolved in consultation with the coauthor.

### Data synthesis and meta-analysis

Included studies will be pooled in a statistical meta-analysis (where possible), using statistical software— R: A language and environment for statistical computing (R Foundation for Statistical Computing, Vienna, Austria). For analysis calculation, effect sizes will be expressed as risk ratios or ORs for dichotomous data or weighted (or standardized) mean differences and 95% CIs for continuous data in this systematic review. If heterogeneity appears across the selected studies after a visual inspection of the forest plot or statistically using the standard χ2 and I2 tests, the choice of the model (random or fixed effects) and method for meta-analysis will be established according to Tufanaru, Munn [25].

### Assessment of publication bias

Data will be reported in the form of tables and figures, if statistical pooling is not possible because of limitations, such as substantial heterogeneity among the included studies. The authors will assess publication bias by generating a funnel plot if over 10 studies are included in a meta-analysis using RevMan V.5.4. In assessing funnel plot asymmetry (where appropriate), statistical tests (Egger test, Begg test, Harbord test) will be performed.

### Assessment of certainty of evidence

The Grading of Recommendations, Assessment, Development and Evaluation (GRADE) approach for assessing the quality of evidence will be followed, and a Summary of Findings (SoF) will be created. The SoF will present the following information, where available: ranking of the quality of the evidence based on the study’s risk of bias, indirectness, inconsistency, imprecision, and risk of publication bias of the review results. The mortality rate and secondary outcomes of all included studies will be included in the SoF table. GRADEpro GDT software (McMaster University, ON, Canada) will be utilized for this purpose.

## Supplementary Information

Supplementary Material 1.

Supplementary Material 2.

Supplementary Material 3.

## Data Availability

The datasets used and/or analyzed during the current study are available from the corresponding author on reasonable request.
