# Supplementary material for: The role of vasopressin in trauma resuscitation: a protocol of a systematic review and meta-analysis of randomized and observational studies
Source: Scand J Trauma Resusc Emerg Med. 2026 Feb 11;34:58. doi: 10.1186/s13049-026-01581-w (PMC12998169; doi:10.1186/s13049-026-01581-w)
Supplement: Supplementary file 1 — Supplementary Material 1. [file 13049_2026_1581_MOESM1_ESM.pdf]

Supplement appendix 1. The search strategies and yielded articles for various resources in the study

**Database (date of search)**	**Search query and method**	**Filters**	**Yielded results**
**PubMed (9/25/2025)**	(("Vasopressins"[Mesh] OR vasopressin[tiab] OR "arginine vasopressin"[tiab] OR AVP[tiab] OR "antidiuretic hormone"[tiab] OR ADH[tiab] OR desmopressin[tiab] OR terlipressin[tiab])) AND (("Wounds and Injuries"[Mesh] OR "Injuries"[tiab] OR trauma[tiab] OR "trauma patient*"[tiab] OR "traumatic injury"[tiab] OR "trauma resuscitation"[tiab]) OR ("Hemorrhagic Shock"[Mesh] OR "Shock, Hemorrhagic"[tiab] OR "hemorrhagic shock"[tiab] OR "massive hemorrhage"[tiab] OR "massive bleeding"[tiab] OR "uncontrolled hemorrhage"[tiab] OR "hemorrhage control"[tiab] OR "exsanguination"[tiab] OR "severe bleeding"[tiab])) AND (	Limited to from 2000 and later, English articles, available full text	373

	("Resuscitation"[Mesh] OR resuscitation[tiab] OR "fluid resuscitation"[tiab]<br>OR "damage control resuscitation"[tiab] OR "blood transfusion"[Mesh]<br>OR transfusion[tiab] OR "massive transfusion"[tiab] OR "shock management"[tiab]<br>OR "critical care"[tiab] OR "intensive care"[tiab])<br>)		
**PubMed central (9/25/2025)**	(<br>vasopressin OR "arginine vasopressin" OR AVP OR "antidiuretic hormone" OR ADH<br>OR desmopressin OR terlipressin<br>)<br>AND<br>(<br>trauma OR "trauma patient*" OR "traumatic injury" OR "trauma resuscitation"<br>OR "hemorrhagic shock" OR "massive hemorrhage" OR "massive bleeding"<br>OR exsanguination OR "damage control resuscitation" OR transfusion<br>)<br>AND<br>(	Limited to from 2000 and later, English articles, available full text	373

	resuscitation OR "fluid resuscitation" OR "blood transfusion"<br>OR "shock management" OR "critical care" OR "intensive care"<br>)		
**Embase (9/25/2025)**	1. 'vasopressin'/exp OR vasopressin:ti,ab OR 'arginine vasopressin':ti,ab<br>OR avp:ti,ab OR 'antidiuretic hormone':ti,ab OR adh:ti,ab<br>OR desmopressin:ti,ab OR terlipressin:ti,ab<br><br>2. 'trauma'/exp OR trauma:ti,ab OR 'trauma patient*':ti,ab OR 'traumatic injury':ti,ab<br>OR 'trauma resuscitation':ti,ab<br><br>3. 'hemorrhagic shock'/exp OR 'hemorrhagic shock':ti,ab OR 'massive hemorrhage':ti,ab<br>OR 'massive bleeding':ti,ab OR exsanguination:ti,ab OR 'damage control resuscitation':ti,ab<br>OR transfusion:ti,ab<br><br>4. 'resuscitation'/exp OR resuscitation:ti,ab OR 'fluid resuscitation':ti,ab<br>OR 'blood transfusion'/exp OR 'blood transfusion':ti,ab	Limited to from 2000 and later, English articles	108

	OR 'shock management':ti,ab OR 'critical care':ti,ab OR 'intensive care':ti,ab<br><br>5. 1 AND (2 OR 3) AND 4		
**Scopus (9/25/2025)**	TITLE-ABS-KEY(<br>vasopressin OR "arginine vasopressin" OR AVP OR "antidiuretic hormone" OR ADH<br>OR desmopressin OR terlipressin<br>)<br>AND TITLE-ABS-KEY(<br>trauma OR "trauma patient*" OR "traumatic injury" OR "trauma resuscitation"<br>OR "hemorrhagic shock" OR "massive hemorrhage" OR "massive bleeding"<br>OR exsanguination OR "damage control resuscitation" OR transfusion<br>)<br>AND TITLE-ABS-KEY(<br>resuscitation OR "fluid resuscitation" OR "blood transfusion"<br>OR "shock management" OR "critical care" OR "intensive care"<br>)	Limited to from 2000 and later, English articles, available full text, completed studies, articles Exclude animal studies, case-reports, narrative reviews	286
**ClinicalTrials.gov (9/25/2025)**	(vasopressin OR "arginine vasopressin" OR AVP OR "antidiuretic hormone" OR ADH OR terlipressin OR desmopressin)<br>AND	Limited to from 2000 and later	135

	(trauma OR "trauma patient" OR "traumatic injury" OR "hemorrhagic shock" OR "massive hemorrhage" OR "damage control resuscitation" OR transfusion OR resuscitation)		
**Cochrane library (9/25/2025)**	([mh "Vasopressins"] OR vasopressin OR "arginine vasopressin" OR AVP OR "antidiuretic hormone" OR ADH OR terlipressin OR desmopressin)<br>AND<br>([mh "Wounds and Injuries"] OR trauma OR "trauma patient*" OR "traumatic injury" OR "trauma resuscitation")<br>AND<br>([mh "Shock, Hemorrhagic"] OR "hemorrhagic shock" OR "massive hemorrhage" OR "massive bleeding" OR exsanguination OR "damage control resuscitation" OR transfusion)<br>AND<br>([mh "Resuscitation"] OR resuscitation OR "fluid resuscitation" OR "blood transfusion" OR "shock management" OR "critical care" OR "intensive care")		28
**Other resources (9/25/2025)**	Forward and backward citation checking of high impact articles* using PaperFetcher online services**	-	339

* [Bauer, S. R., Sacha, G. L., Lam, S. W., Wang, L., Reddy, A. J., Duggal, A., & Vachharajani, V. (2022). Hemodynamic Response to Vasopressin Dosage of 0.03 Units/Min

vs. 0.04 Units/Min in Patients With Septic Shock. Journal of intensive care medicine, 37(1), 92–99. https://doi.org/10.1177/0885066620977181],

(NOTE: Studies addressing non-traumatic shock (e.g., septic shock) were used solely as citation sources and were not considered for eligibility.)

[Sims CA, Holena D, Kim P, Pascual J, Smith B, Martin N, Seamon M, Shiroff A, Raza S, Kaplan L, Grill E, Zimmerman N, Mason C, Abella B, Reilly P. Effect of Low-Dose Supplementation of Arginine Vasopressin on Need for Blood Product Transfusions in Patients With Trauma and Hemorrhagic Shock: A Randomized Clinical Trial. JAMA Surg. 2019 Nov 1;154(11):994-1003. doi: 10.1001/jamasurg.2019.2884. PMID: 31461138; PMCID: PMC6714462.],

[Cohn, S. M., McCarthy, J., Stewart, R. M., Jonas, R. B., Dent, D. L., & Michalek, J. E. (2011). Impact of low-dose vasopressin on trauma outcome: prospective randomized study. World journal of surgery, 35(2), 430–439. https://doi.org/10.1007/s00268-010-0875-8], and

[Voelckel W. G. (2025). Vasopressin in traumatic hemorrhagic shock. Current opinion in anaesthesiology, 38(2), 81–92. https://doi.org/10.1097/ACO.0000000000001456]

** Pallath, A., & Zhang, Q. (2022). Paperfetcher: A tool to automate handsearching and citation searching for systematic reviews. Research Synthesis Methods. DOI: 10.1002/jrsm.1604
