# Supplementary material for: The role of vasopressin in trauma resuscitation: a protocol of a systematic review and meta-analysis of randomized and observational studies
Source: Scand J Trauma Resusc Emerg Med. 2026 Feb 11;34:58. doi: 10.1186/s13049-026-01581-w (PMC12998169; doi:10.1186/s13049-026-01581-w)
Supplement: Supplementary file 2 — Supplementary Material 2. [file 13049_2026_1581_MOESM2_ESM.pdf]

Supplement Appendix 2. Data extraction sheet

Identification

First author

Year

Country

Journal

DOI

Funding (Y/N)

Conflict of interest (Y/N)

Study characteristics

Study design (RCT, cohort, case-control, before–after, registry)

Setting (prehospital, ED, OR, ICU, mixed)

Recruitment years

Sample size (per arm, total)

Inclusion criteria

Exclusion criteria

Population

Age (mean ± SD, median [IQR])

Sex distribution (M/F, %)

Baseline injury severity (ISS, GCS, SBP, shock index)

Mechanism of trauma (blunt, penetrating, mixed)

Pre-intervention mortality risk (if reported)

Intervention

Intervention drug: vasopressin / terlipressin / desmopressin

Dose (bolus / infusion rate, units per min)

Route (IV, intraosseous)

Timing relative to injury (prehospital, ED, intra-op, ICU)

Duration of therapy

Co-interventions (fluid resuscitation, blood products, TXA, REBOA, MTP protocol)

Comparator

Placebo / standard care / norepinephrine / epinephrine / other

Dose and regimen if drug comparator

Usual resuscitation protocol applied? (Y/N, describe)

Outcomes

Primary (Mortality)	Mortality type (in-hospital, 24h, 48h, 30d, ICU, cause-specific)<br>Numerator: deaths per arm<br>Denominator: total per arm<br>Time-to-event data (HR, SE, CI, p-value)<br>Adjusted effect estimate (yes/no, covariates included)
Secondary (as available)	Hemodynamic response (MAP, HR, lactate, shock index)<br>Fluid/blood product requirements (total crystalloids, blood units, plasma, platelets, ratios)<br>Vasopressor sparing effect (yes/no, dose reduction)<br>Organ failure scores (SOFA, MODS)<br>Length of stay (ICU, hospital)<br>Adverse events (ischemia, arrhythmia, hyponatremia, renal injury, limb necrosis)

Risk of Bias / Study Quality

Randomization described (Y/N)

Allocation concealment (Y/N)

Blinding (participants, personnel, outcome assessors)

Completeness of outcome data (attrition %)

Selective reporting (Y/N)

Other bias (e.g., baseline imbalance, early stopping, industry sponsorship)

Overall RoB (low / some concern / high for RCTs; ROBINS-I domains for observational)

Notes

Author contact attempted (Y/N, date)

Protocol registration available? (Y/N, registry number)

Comments (special considerations, subgroup notes, deviations)
